# Comparison of pre-chop technique using a reverse chopper and classic stop-and-chop technique in the treatment of high myopia associated with nuclear cataract

**DOI:** 10.1186/s12893-022-01658-0

**Published:** 2022-05-28

**Authors:** Ke Yang, Jiaxin Li, Weihua Zhang, Zhanjiang Liu, Chenjie Song, Yang Zhao

**Affiliations:** 1 Beijing Tongren Eye Center, Beijing Key Laboratory of Ophthalmology and Visual Science, Beijing Tongren Hospital, Capital Medical University, Dongjiaominxiang 1st, Dongcheng District, Beijing, 100730 China; 2 Medical School, Pingdingshan University, Pingdingshan, 467000 China; 3 Liaocheng People’s Hospital, Liaocheng, 252000 China; 4 Chaoyang Central Hospital, Chaoyang City, 122000 Liaoning Province China; 5 Mei’ermu Hospital, Beijing, China

**Keywords:** Cataract extraction, High myopia, Phacoemulsification, Pre-chop technique, Reverse chopper, Stop-and-chop technique

## Abstract

**Background:**

To evaluate the safety and efficacy of the pre-chop technique using a novel reverse chopper vs. the classic stop-and-chop technique in phacoemulsification for patients with high myopia and associated grade III–IV nuclear cataracts.

**Methods:**

In this prospective cohort study, a total of 44 consecutive patients (44 eyes) with grade III–IV nuclear cataracts who were admitted to our hospital for cataract surgery between March 2018 and September 2018 were enrolled. All patients had ocular axial length > 27 mm and myopic refraction more than -10 diopters. Patients were randomly divided into a pre-chop group and stop-and-chop group using a randomization table. Nucleus splitting was performed surgically in both groups using either the pre-chop technique with reverse chopper or the classic stop-and-chop technique.

**Results:**

Postoperative visual acuity was significantly improved in both groups compared with preoperative values. Significantly better visual acuity, lower degree of corneal edema and lower rates of corneal endothelial cell loss were observed in the pre-chop group compared to those in the classic stop-and-chop group. No complications were reported in either group.

**Conclusions:**

In treating patients with high myopia associated with grade III–IV cataracts, the pre-chop technique using a reverse chopper reduces damage to corneal endothelial cells and improves visual acuity better than the classic stop-and-chop technique.

**Supplementary Information:**

The online version contains supplementary material available at 10.1186/s12893-022-01658-0.

## Background

The prevalence of myopia and high myopia is increasing globally, especially in Eastern and Southern Asia [1]. The pathological conditions associated with high myopia, including retinal damage, cataracts, and glaucoma, significantly increase the risk of visual impairment [2]. High myopia is defined as myopia with ≥ 27.00 mm of ocular axial length and more than − 10.00 diopters of refraction [3]. Rates of myopia with ≥ 27.0 mm of the ocular axial length among elderly Chinese are reported to be significantly greater than 1.0%, compared to the rate of 0.1% reported in European and American populations [4–6]. Patients with high myopia usually have associated hard nuclear cataracts, and therefore will usually undergo early cataract surgery [7].

The nucleus-splitting technique is one of the key aspects of cataract surgery and requires surgeons to control ultrasound and negative pressure, as well as hand–foot coordination, together. These increase the technical difficulty of the procedure. When compared with conventional ultrasound phacoemulsification, it reduces required ultrasound energy, surgery time, extent of tissue damage, and occurrence of complications associated with energy release [8]. Consequently, stop-and-chop phacoemulsification is widely used by many surgeons [9]. In patients with high myopia and associated cataracts, the anterior chamber is deep and the nucleus of the lens is relatively hard; thus, the phacoemulsification procedure often induces breakage of the posterior capsule [10]. Therefore, despite wide use among surgeons, phacoemulsification surgery is classified as a highly difficult surgery [11].

The manual pre-chop technique reduces the energy and time required for intraoperative phacoemulsification and reduces damage to the intraocular tissue [12], which is shown to be useful in both normal and small pupil cataract surgery [13]. However, most pre-chop methods require downward pressure on the lens, which may result in a certain amount of traction on the suspensory ligaments [14]. For this reason, these methods provide only poor management of the hard nucleus [15]. These shortcomings limit the application of new techniques in the treatment of myopia associated with cataracts. Further, femtosecond laser nuclear pre-chopping also manages the hard nucleus poorly and requires expensive equipment, limiting its wider use [16].

Given the above-mentioned limitations, we developed a novel pre-chop technique using a reverse chopper before phacoemulsification of the lens nucleus, which achieves efficient in situ nucleus-splitting without using ultrasonic energy. Our new technique exhibits advantages such as reducing time and energy during phacoemulsification, as well as reducing surgical complications. We have applied this technique in surgeries for high myopia [17] and complex cataracts [18]. The present study was conducted to compare clinical outcomes between the new pre-chop technique and the classic stop-and-chop technique in patients with myopia and associated cataracts.

## Patients and methods

### Study design and sample

A prospective cohort study design was adopted. Consecutive patients with high myopia and associated cataracts who were admitted to the Cataract Center of Beijing Tongren Hospital between March and September 2018 were enrolled. The inclusion criteria were: (1) patients with grade III–IV nuclear cataract based on Emery’s classification [19]; (2) an ocular axial length > 27 mm, and myopic refraction more than − 10 diopters; (3) no obvious or only mild lens tremor on mydriatic examination, and no history of glaucoma or iritis; and (4) patients had shown good compliance, knew that new surgical equipment would be used in the surgery, were aware of the risks of surgery, agreed to participate in the study, provided signed informed consent, and agreed to be followed regularly. The exclusion criteria were: (1) patients with medical history of diabetic retinopathy, macular edema, pseudoexfoliation, or optic neuropathy; (2) patients who had undergone a specific ocular surgery within the preceding 60 months; and (3) patients with a history of eye trauma.

Patients were randomly divided into a pre-chop group and a stop-and-chop group using a randomization table. Before surgery, slit-lamp microscopic examination was performed for all patients and showed a clear cornea, a deep anterior chamber, and normal iris texture without iris atrophy or depigmentation. After mydriasis, the lens was observed to be in a normal location, the nucleus became turbid, the color of the nucleus was dark yellow, brown or amber, and the intraocular pressure was ≤ 21 mm Hg (noncontact tonometer TX-20; Canon).

### Ethical considerations

The study protocol was reviewed and approved by the Institutional Review Board of Beijing Tongren Hospital (TRECKY2017-028). All included patients provided signed informed consent to participate in the study.

### Surgical procedure

**Fig. 1:**
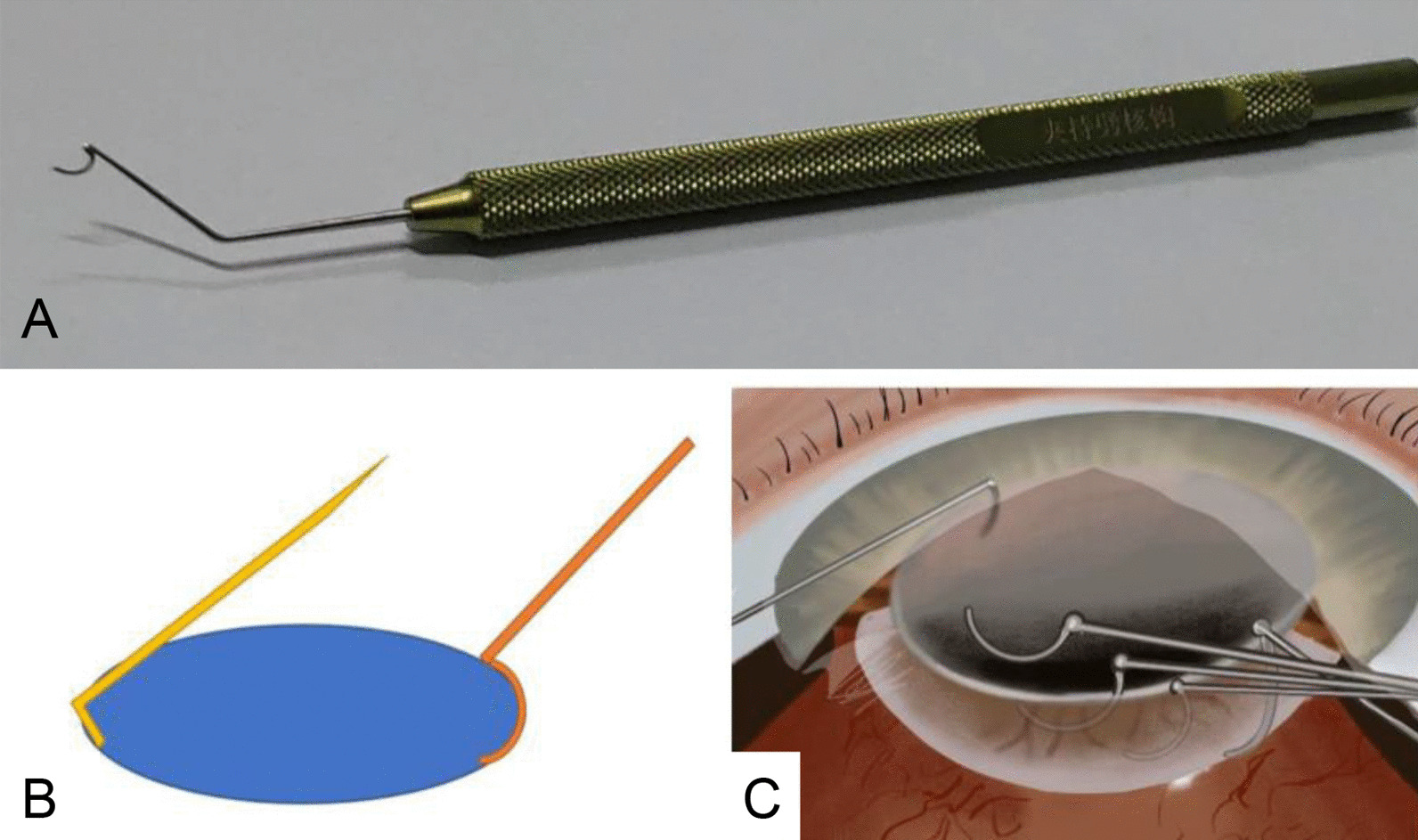
Devices for pre-chop technique using a reverse chopper. **A** A reverse chopper. **B** The meridional section of the lens shows the locations of the two choppers. **C** The trajectory of the lower hook holding the nuclear splitting hook

A specially made metal reverse chopper is used during the surgery (Fig. 1A). This chopper is shaped like an inverted circular arc with a round and blunt tip and an inner blade on the circular arc. The distance between the 2 tips of the arc is about 3 mm. The distal curvature is slightly larger than the proximal curvature (Chinese National Patent Number 201520394994X) [20]. All surgeries in this study were performed by the same experienced surgeon (Y. Zhao).

#### Pre-chop phacoemulsification and intraocular lens implantation

The procedure followed our previous report with some modifications [20]. In detail, phacoemulsification and intraocular lens (IOL) implantation are carried out using an ultrasound emulsification device (INFINITI Vision System; US Alcon, Fort Worth, TX, USA). The intraoperative phacoemulsification parameters are: ultrasound energy 55%, negative pressure 380 mm Hg (1 mm Hg = 0.133 kPa), and a flow rate of 40 mL/min. A standard 3.0-mm incision is made at the 10 o’clock position of the transparent cornea. Ocular viscoelastic solution (EYEFILL^®^S.C, Bausch & Lomb, Montpellier, France) is injected into the anterior chamber. A standard puncture opening is made at the 2 o’clock position using a 15° puncture scalpel. Continuous curvilinear capsulorhexis is performed for the anterior capsule of the lens using capsulorhexis forceps. The diameter of the capsule opening is 5–6 mm.

The reverse chopper is inserted into the anterior chamber via the incision made at the 10 o’clock position of the cornea. Although it is a standard 3.0-mm incision, the operatable incision can be as small as 2.2 mm in width. The distal end of the chopper is then gently pressed downward to contact the nucleus of the lens. The reverse chopper is then tilted laterally and slid into the capsule along the nucleus of the lens. The chopper is then raised gradually to bury its arcuate part into the cortical shell located between the nucleus of the lens and the capsule. Its arc-shaped inner blade is placed perpendicular to the nucleus equator and fixed.

A Nagahara chopper is inserted into the anterior chamber via the lateral incision made at the 2 o’clock position. The Nagahara chopper is slid into the space beneath the cortex and the capsule membrane so that its blade is perpendicular to the equator of the nucleus. The surgeon holds the Nagahara chopper in the left hand, and the reverse chopper in the right hand, which is placed on the radial line of the lens (Fig. 1B, C). The two devices are pushed toward the center of the lens, and the surgeon ensures that both devices move in the horizontal direction. The nucleus of the lens is split, and the Nagahara chopper meets the reverse chopper at the center of the lens. The two devices are then gently separated horizontally to divide the nucleus completely into 2 semi-ellipsoids (Additional file 1: Fig. S1). Then the reverse chopper remains near the right half of the nucleus while the Nagahara chopper is slid again into the bottom of the capsule at the 8 o’clock position. It is then pulled to the center to divide the right half of the nucleus into 2 parts (Additional file 2: Fig. S2).

Next, a conventional phacoemulsification procedure is carried out. The phacoemulsification needle is inserted, and the nucleus is suctioned to the iris plane. The nucleus is removed after emulsification. The cortex of the lens is removed, and an IOL (AcrySof IQ Aspheric Natural IOL SN60WF, Alcon, Vernier-Geneva, Switzerland) is implanted in the capsule. In some patients, a capsular tension ring is also implanted. After surgery, tobramycin dexamethasone eye ointment is applied to the conjunctival sac.

#### Stop-and-chop phacoemulsification and intraocular lens implantation

The preoperative preparation, equipment, surgical incision, circular capsulorhexis, and postoperative procedures for the stop-and-chop method are similar to those previously reported for the new procedure. Hydrodissection is performed after continuous curvilinear capsulorhexis. The phacoemulsification needle is then used to suction the nucleus to its tip. The suctioned nucleus is then split using the chopper, and phacoemulsification is performed. The nucleus is gradually broken into pieces and removed. Next, the cortex of the lens is removed, and an IOL is implanted into the capsule. In some patients, a capsular tension ring was implanted.

### Observation indicators and evaluation standard

The actual ultrasound energy (%) and valid ultrasound time (s) required for the phacoemulsification of the lens nucleus were compared between the two groups in this study. Best corrected visual acuity (BCVA) was recorded before surgery and then at postoperative days 1 and 7. A corneal endothelial cell count was carried out before surgery and at 1 month after surgery using a specular microscope (SP-3000P; Topcon Corp, Tokyo, Japan). The status of corneal edema and postoperative complications were compared between groups at postoperative days 1 and 7. Corneal edema and opacity were classified using Dickey’s standard 5: Grade 0: the cornea is transparent; grade 1: the cornea is slightly opaque (hazy); grade 2: the cornea is opaque, but the structures of the anterior chamber are clearly visible; grade 3: the corneal opacity is aggravated, and observation of the anterior chamber is difficult; grade 4: corneal opacity is severe, the iris structure cannot be observed, and the anterior chamber is not visible.

### Statistical analysis

All data were analyzed using SPSS version 19.0 (IBM Corp., Armonk, NY, USA). Based on results normality testing, normally distributed data are represented as $\overline{x }$ ± *s* (mean ± standard deviation, SD)*,* skewed data are represented as *M* (*Q1, Q3*), and frequency data are represented as the number of eyes. A single-factor, completely randomized design was used for grouping. An independent 2-sample *t*-test was used to compare the ultrasound energy and valid ultrasound time between the pre-chop group and the stop-and-chop group. The Chi-square test was used to compare visual acuity and corneal edema between the pre-chop group and the stop-and-chop group. An independent 2-sample *t*-test was used to compare the postoperative corneal endothelial cells and the loss rate between the pre-chop group and the stop-and-chop group. All tests were 2-tailed and a significance level of 0.05 was adopted.

## Results

### Patients’ baseline characteristics

A total of 44 patients (44 eyes) with high myopia and associated cataracts were enrolled, and 22 patients (22 eyes) were randomly placed into each of two groups: a pre-chop group and a stop-and-chop group. Patients’ ages ranged overall from 37 to 68 years. In the pre-chop group, 11 eyes were in males and 11 in females, mean age was 57.41 years (range 38–68 years), and the mean with standard deviation (mean ± SD) preoperative BCVA was 0.14 ± 0.09. In the stop-and-chop group, 10 eyes were in males and 12 in females, the mean age was 56.36 years (range 37–67 years), and the mean preoperative BCVA was 0.15 ± 0.08. No significant differences were noted between the 2 groups in sex, age and BCVA (Table 1). Surgeries were performed successfully in both groups, and no serious intraoperative complications occurred in either group.

**Table 1:** Patients’ preoperative baseline characteristics by group

	Pre-chop group	Stop-and-chop group	p-value ^a^
Eye (n)	22	22	
Age	57.41 ± 8.18	56.36 ± 8.43	0.679
Gender			
Male	11 (50.0%)	10 (45.5%)	0.763^b^
Female	11 (50.0%)	12 (54.5%)	
Ocular axial length	29.29 ± 0.28	29.46 ± 0.27	0.678
BCVA	0.14 ± 0.09	0.15 ± 0.08	0.888
Astigmatism	1.64 ± 0.11	1.63 ± 0.10	0.939
Difference in Corneal edema score	1.41 ± 0.50	1.41 ± 0.50	1.000
Endothelial cell count 1 month after surgery	2079.67 ± 163.87	2019.09 ± 245.70	0.341

*BCVA* best corrected visual acuity

^a^Independent t test

^b^Chi-square test

### Comparison of intraoperative ultrasound energy and time between groups

The mean actual ultrasound energy used in the pre-chop group (55.00 ± 0.00) was the same as in the stop-and-chop group (55.00 ± 0.00). The mean ultrasound time in the pre-chop group was significantly shorter than that in the stop-and-chop group (48.55 ± 11.62 vs. 56.68 ± 14.23, *P* = 0.04).

### Comparison between preoperative and postoperative BCVA

The postoperative BCVA for visual acuity was significantly improved in both groups compared with the preoperative values (Table 2). No significant differences in preoperative BCVA were found between the two groups (*P* = 0.888). The BCVA was significantly improved on postoperative day 1 in the pre-chop group (0.32 ± 0.13 from 0.14 ± 0.09; *P* < 0.001), and in the stop-and-chop group (0.22 ± 0.13 from 0.15 ± 0.08; *P* = 0.004). On postoperative day 7, the mean BCVA in the pre-chop and stop-and-chop groups had improved to 0.58 ± 0.14 (*P* < 0.001) and to 0.49 ± 0.15 (*P* < 0.001), respectively. Postoperative mean BCVA was significantly higher in the pre-chop group than that in the stop-and-chop group (*P* = 0.011 on postoperative day 1, *P* = 0.032 on day 7).

**Table 2:** Differences between preoperative and postoperative BCVA values by group

	Pre-chop group	Stop-and-chop group	p-value^a^
Eye (n)	22	22	
A. Preoperative BCVA	0.14 ± 0.09	0.15 ± 0.08	0.888
B. On postoperative day 1	0.32 ± 0.13	0.22 ± 0.13	0.011
C. On postoperative day 7	0.58 ± 0.14	0.49 ± 0.15	0.032
Difference between A and B	0.18 ± 0.11	0.07 ± 0.11	0.002
*p*-value (A vs. B)^b^	< 0.0001	0.004	
Difference between A and C	0.44 ± 0.12	0.34 ± 0.13	0.012
*p*-value (A vs. C)^b^	< 0.0001	< 0.0001	
Difference between B and C	0.26 ± 0.15	0.27 ± 0.12	0.826
*p*-value (B vs. C)^b^	0.0001	0.0001	

*BCVA* best corrected visual acuity

^a^Difference between two groups (Pre-chop group and Stop-and-chop group), used independent t test

^b^Difference between preoperative and postoperative BCVA using paired Student’s T test

^a^Independent t test

### Comparison of postoperative corneal edema scores

Varying degrees of corneal edema were observed one day after surgery and resolved gradually with treatment (medication: tobramycin dexamethasone eyedrops were used 6 times/day for 2 weeks). The degree of corneal edema in the pre-chop group was significantly lower than that in the stop-and-chop group on postoperative days one (*P* = 0.020) and 7 (*P* = 0.013) (Table 3).

**Table 3:** Comparison of the number of eyes with varying degrees of corneal edema by group

	Pre-chop group	Stop-and-chop group	p-value ^a^
Eye (n)	22	22	
One day after surgery			
Grade 0	0 (0.00%)	2 (9.09%)	0.020
Grade 1	5 (22.72%)	2 (9.09%)	
Grade 2	13 (59.09%)	6 (27.27%)	
Grade 3	4 (18.18%)	12 (54.54%)	
Seven days after surgery			
Grade 0	10 (45.45%)	4 (18.18%)	0.013
Grade 1	12 (54.54%)	10 (45.45%)	
Grade 2	0 (0.00%)	7 (31.82%)	
Grade 3	0 (0.00%)	1 (4.55%)	

^a^Chi-square test

### Comparison of corneal endothelial cell status between groups

Prior to surgery, no significant differences were noted in the number of corneal endothelial cells between the two groups (*P* = 0.174). One month after surgery, the number of corneal endothelial cells in the pre-chop group was slightly higher than that in the stop-and-chop group, but without significant differences (*P* = 0.341). However, the rate of corneal endothelial cell loss in the pre-chop group was significantly lower than that in the stop-and-chop group one month after the surgery (*P* = 0.001) (Table 4).

**Table 4:** Comparison of corneal endothelial cells status at preoperative and 1 month postoperative by group

	CEC^a^ count		
	Pre-chop group	Stop-and-chop group	p-value ^b^
Eye (n)	22	22	
At preoperative	2333.45 ± 161.55	2414.91 ± 163.62	0.104
One month postoperative	2079.67 ± 163.87	2019.09 ± 245.70	0.341
Rate of CEC loss (%)	10.87 ± 0.78	16.55 ± 1.37	0.001

^a^*CEC* corneal endothelial cells

^b^Independent t test

## Discussion

The present study has shown that the new pre-chop technique using a reverse chopper is a safe and efficient technique in treating patients with high myopia associated with grade III–IV cataracts. Compared with patients in the classic stop-and-chop group, those in the pre-chop group had significantly less corneal edema, lower corneal endothelial cell loss rate and better BVCA postoperatively. Our new pre-chop technique displayed better recovery of visual acuity and reduced damage to corneal endothelial cells compared to the classic stop-and-chop approach, suggesting that it can be used safely, effectively and quickly in treating patients with high myopia and associated hard nuclear cataracts, while greatly reducing the application of phacoemulsification energy and decreasing the overall difficulty of the surgery.

Various techniques have been developed for cataract surgery in order to reduce the damages to the capsule. Technique like miloop does not require concurrent ultrasound [21, 22], which seems to be a very good option. But there was a trend towards lower endothelial cell loss in the miLOOP eyes [21], and miloop technique needs specialized tools and longer time for operation and familiar with. Thus, we prefer pre-chop with lower ultrasound usage than miloop.

Recent study has suggested that directing ultrasound at the capsule is more suitable for this type of cataract surgery than directing ultrasound inside the capsule, primarily because of the significantly deeper anterior chambers in patients with high myopia [20]. However, although the ultrasound energy required in the two types of surgery is similar, the pre-chop technique significantly reduces intraoperative complications such as capsule breakage, suspensory ligament rupture, and others. Recently, several new manual pre-chop techniques, including the cystotome-assisted pre-chop technique, the pinpoint pre-chop technique, and the use of a reverse chopper with the pre-chop technique have been developed specifically to help reduce the energy and time requirements of intraoperative phacoemulsification, including decreasing damage to eye tissue, reducing the loss of corneal endothelium, and accelerating the speed of postoperative recovery [17]. The effectiveness of these techniques has been demonstrated in routine cataract surgery [18]. In fact, our eye surgery group is planning to broaden the application of the pre-chop technique using a reverse chopper to treat patients with other types of complicated cataracts.

In the present study, we found that the force applied when using the reverse chopper in the right hand during the pre-chop procedure, and the force applied by the Nagahara chopper in the left hand must be on the diagonal line in the horizontal direction to carry out in situ nucleus splitting, and no downward pressing force is applied to the lens nucleus. This technique is shown to reduce traction on the suspensory ligament, avoid breakage of the suspensory ligaments, and increase the surgical success rate [23], making it especially suitable for patients with high myopia associated with cataracts. Importantly, because the pre-chop technique reduces both the ultrasound energy and time required, the overall quality of cataract surgery is improved. Recently, we developed a novel pre-chop procedure using a reverse chopper that can be embedded in the equator of the lens so that the force can be applied on the horizontal direction of the lens to carry out in situ pre-chopping of the nucleus that only rarely pulls on the suspensory ligaments; this pre-chop technique significantly reduces the intraoperative use of ultrasound energy, in turn reducing the loss of corneal endothelium and injuries to other intraocular structures [24].

In the present study, we applied the pre-chop technique using a reverse chopper together with the Nagahara chopper, placing them at the 5 o’clock position and 11 o’clock position in the capsule; then, we held and fixed the lens nucleus, applying force to the center of the nucleus on a diagonal line in the horizontal direction, and divided the nucleus into two parts as if using scissors. Because the suspensory ligament is loose in myopia patients, the nucleus is not rotated 90° after being divided into two parts. The reverse chopper remains close to the right half of the lens nucleus while the Nagahara chopper is slid into the 8 o’clock position of the equator, and then the right half of the nucleus is split into two parts using the reverse chopper. Finally, the entire nucleus is divided into three free pieces. During the whole process of nucleus splitting, no ultrasound energy is released, which greatly reduces the valid ultrasound time, and provides satisfactory outcomes. Both the reverse chopper and the Nagahara chopper have inner blades that fix the nucleus of the lens tightly during nucleus splitting, in which the relatively hard lens nuclei can be split easily. Moreover, the round and blunt design of the tip effectively protects the membrane of the posterior capsule during nucleus splitting and rotation. The most difficult step of the surgery is nucleus splitting. The nucleus should be split safely and quickly into 2–4 pieces for removal from the capsule. Such safe and quick nucleus splitting is the most important step for a successful surgery. However, during the surgery in patients with high myopia with cataracts, the anterior chamber is abnormally deepened, and the suspensory ligament is loose. Therefore, whether the surgeon uses a manual pre-chop technique or a stop-and-chop technique, the operating space is limited when the downward force is applied to the lens nucleus, and the downward force must be gently controlled to avoid suspensory ligament rupture due to excessive traction.

In conclusion, the reverse chopper can be used safely and effectively during the pre-chop procedure for patients with high myopia and associated hard nuclear cataracts, greatly reducing the application of phacoemulsification energy and decreasing the difficulty of surgery. Applying the present pre-chop technique using a reverse chopper displays better recovery of visual acuity and greater reduction of damage to corneal endothelial cells compared to the classic stop-and-chop approach. We have applied the newly developed technique to the treatment of difficult cataracts and obtained favorable outcomes, including treating patients with cataract grade IV hard nucleus and small pupil cataracts [17, 18]. Given the instrument’s simplicity, and its efficient use of time and short learning curve, the reverse chopper has the potential to be used widely.

## Supplementary Information

**Additional file 1: Figure S1.** A The reverse chopper was placed into the anterior chamber horizontally, and the distal end of the chopper was gently pressed downward and slid into the space between the capsule and the equator. B The reverse chopper was held erectly to bury its arcuate part in the cortical shell located between the nucleus of the lens and the capsule, and its arc-shaped inner blade was placed perpendicular to the nucleus equator. The two devices were pushed toward the center of the lens, and the surgeon ensured that both devices were moved in the horizontal direction to split the nucleus of the lens. C The Nagahara chopper met the reverse chopper at the center of the lens. The two devices were then gently separated horizontally to divide the nucleus completely into two semi-ellipsoids.

**Additional file 2: Figure S2.** A The reverse chopper remained near the right half of the nucleus, while the Nagahara chopper was slid again into the bottom of the capsule at the 8 o’clock position. B The Nagahara chopper was pulled to the center to divide the right half of the nucleus into two parts.

## Data Availability

The datasets used during the current study are available from the corresponding author on reasonable request.

## Abbreviations

BCVA: Best corrected visual acuity
CEC: Corneal endothelial cells
