# Supplementary figures and images for: Comparison of pre-chop technique using a reverse chopper and classic stop-and-chop technique in the treatment of high myopia associated with nuclear cataract
Source: BMC Surg. 2022 May 28;22:206. doi: 10.1186/s12893-022-01658-0 (PMC9148448; doi:10.1186/s12893-022-01658-0)

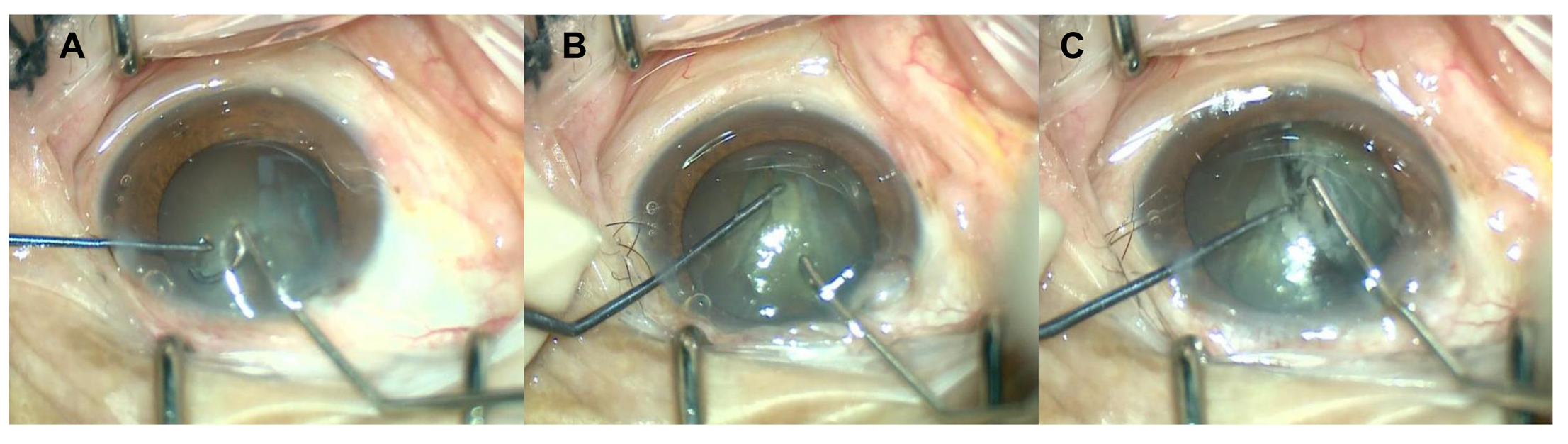

Supplement: Supplementary file 1 — Additional file 1: Figure S1. A The reverse chopper was placed into the anterior chamber horizontally, and the distal end of the chopper was gently pressed downward and slid into the space between the capsule and the equator. B The reverse chopper was held erectly to bury its arcuate part in the cortical shell located between the nucleus of the lens and the capsule, and its arc-shaped inner blade was placed perpendicular to the nucleus equator. The two devices were pushed toward the center of the lens, and the surgeon ensured that both devices were moved in the horizontal direction to split the nucleus of the lens. C The Nagahara chopper met the reverse chopper at the center of the lens. The two devices were then gently separated horizontally to divide the nucleus completely into two semi-ellipsoids. [file 12893_2022_1658_MOESM1_ESM.tif]

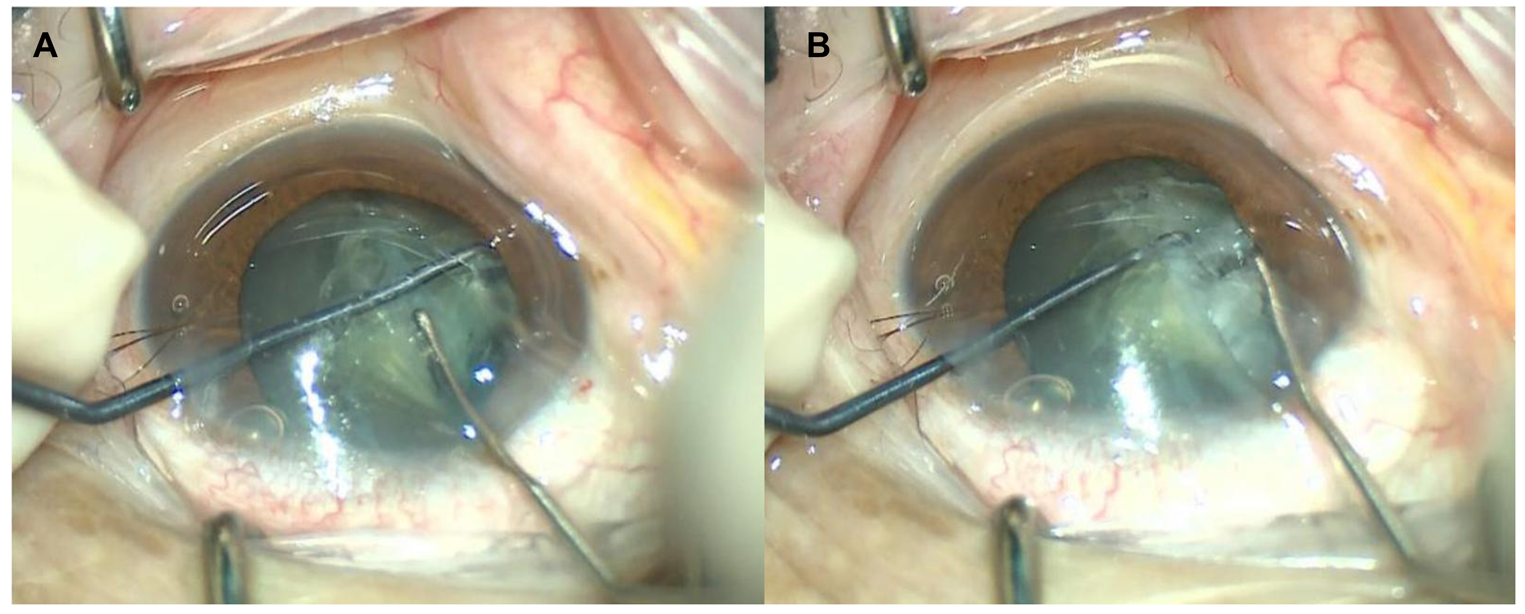

Supplement: Supplementary file 2 — Additional file 2: Figure S2. A The reverse chopper remained near the right half of the nucleus, while the Nagahara chopper was slid again into the bottom of the capsule at the 8 o’clock position. B The Nagahara chopper was pulled to the center to divide the right half of the nucleus into two parts. [file 12893_2022_1658_MOESM2_ESM.tif]
